# Combined Influences of Model Choice, Data Quality, and Data Quantity When Estimating Population Trends

**DOI:** 10.1371/journal.pone.0132255

**Published:** 2015-07-15

**Authors:** Pamela Rueda-Cediel, Kurt E. Anderson, Tracey J. Regan, Janet Franklin, Helen M. Regan

**Affiliations:** 1 Department of Biology, University of California Riverside, Riverside, CA, United States of America; 2 Arthur Rylah Institute for Environmental Research, Heidelberg, Victoria, Australia; 3 The School of Biosciences, The University of Melbourne, Parkville, Victoria, Australia; 4 School of Geographical Sciences and Urban Planning, Arizona State University, Tempe, AZ 85287, United States of America; Tulane University Health Sciences Center, UNITED STATES

## Abstract

Estimating and projecting population trends using population viability analysis (PVA) are central to identifying species at risk of extinction and for informing conservation management strategies. Models for PVA generally fall within two categories, scalar (count-based) or matrix (demographic). Model structure, process error, measurement error, and time series length all have known impacts in population risk assessments, but their combined impact has not been thoroughly investigated. We tested the ability of scalar and matrix PVA models to predict percent decline over a ten-year interval, selected to coincide with the IUCN Red List criterion A.3, using data simulated for a hypothetical, short-lived organism with a simple life-history and for a threatened snail, *Tasmaphena lamproides*. PVA performance was assessed across different time series lengths, population growth rates, and levels of process and measurement error. We found that the magnitude of effects of measurement error, process error, and time series length, and interactions between these, depended on context. We found that high process and measurement error reduced the reliability of both models in predicted percent decline. Both sources of error contributed strongly to biased predictions, with process error tending to contribute to the spread of predictions more than measurement error. Increasing time series length improved precision and reduced bias of predicted population trends, but gains substantially diminished for time series lengths greater than 10–15 years. The simple parameterization scheme we employed contributed strongly to bias in matrix model predictions when both process and measurement error were high, causing scalar models to exhibit similar or greater precision and lower bias than matrix models. Our study provides evidence that, for short-lived species with structured but simple life histories, short time series and simple models can be sufficient for reasonably reliable conservation decision-making, and may be preferable for population projections when unbiased estimates of vital rates cannot be obtained.

## Introduction

Estimating and projecting population trends is essential to ascertaining threat status and developing conservation strategies. For example, the IUCN Red List Categories and Criteria, the Convention on the International Trade in Endangered Species, the NatureServe Conservation Ranks, Partners in Flight, and the Committee on the Status of Endangered Wildlife in Canada all use protocols that rely on population trends as extinction risk surrogates [1]. A widely used tool to assess and project population trends is population viability analysis (PVA). This technique is founded on the principles of risk assessment, using population models to estimate risk of decline or extinction within a specified time frame [2,3].

PVA has enjoyed broad use for conservation decision-making. The IUCN Red List Categories and Criteria, for example, include criteria that rely on quantitative analyses of the probability of extinction and recommend PVA to estimate percent declines and extinction probabilities [4]. PVA has also been used to inform decisions to list species under the United States Endangered Species Act including the northern spotted owl (*Strix occidentalis caurina*), and is commonly used to identify conservation management strategies, for recovery planning, and for habitat conservation plans for threatened species [5,6,7]. Despite their long-standing use, the question of how to construct and apply PVAs is still an area of active research and debate, especially under the high environmental variability and data uncertainty that characterize most conservation applications [5,8].

Several types of population models have been implemented in PVA with the choice typically based on the available data and species life history [9,10]. The most common types of data used to parameterize population models are time series data, i.e. population counts taken on a regular or semi-regular basis. Estimated viability depends critically on both the quality and quantity of these data [9,11]. The length of the time series, environmental variability, and measurement error, in conjunction with the type of population model used, can all influence the bias and precision of projections generated by PVA [10,12,13]. While time series length should be commensurate with the degree of variability in population counts, it is unknown what the time series length should be for different levels of variability and how this is affected by measurement error and model choice.

Theoretical and empirical studies have established that extinction risk estimates can be strongly influenced by environmental stochasticity (i.e. process error; [14,15]). In general, high levels of environmental stochasticity increase the risk of extinction [16,17]. Variability in population estimates is exacerbated by measurement error, i.e. uncertainty in population counts due to inexact observations. This error can result in overly pessimistic risk estimates if subsumed into vital rate variability. While state-space models can disentangle measurement error from process error (e.g. [18]), high technical and data demands present significant barriers to their widespread use.

Time series length can potentially have a greater effect on risk estimates than measurement error [13]. For instance, Hovestadt and Nowicki [19] found population declines derived from short time series to be overestimated with scalar models that included density dependence. Additionally, Humbert et al. [20] showed that state space models produced larger confidence intervals around population trend estimates for shorter time series and recommended a minimum of 10 years of data for reliable extinction risk estimates. However, these conclusions will depend on the type of population model used to generate risk estimates, how these models are parameterized, and how much measurement error and variability are present in the data.

While there are many types of PVA model structures, they generally fall within two main categories [21]: scalar (count-based) or matrix (demographic) models. The choice of model structure often depends on the level of aggregation across life history stages evident in empirical data. Scalar PVAs are based on counts of individuals that are assumed to be identical which may not reflect the real structure of the population. Matrix PVAs, on the other hand, assume individuals differ in their contributions to population growth, categorizing them according to size, age or stage. This makes matrix models potentially more applicable to management because they allow for evaluation of management options for specific life stages or traits (e.g. [22]), which can have significant consequences. For example, Dunham et al. [23] found that population projections generated with scalar models tended to overestimate the risk of decline observed in a simulated age-structured population. The bias, caused by a misattribution of fluctuations due to age structure to environmental variability, was found to be exacerbated by increased generation length. However, matrix model structure also subjects them to greater uncertainty, as more parameters (and therefore more data) are required [24]. Parameterization of matrix models from limited or error-laden census data can present many challenges relative to scalar models. Thus, scalar models are often preferred—despite the greater perceived realism of matrix models—because their data requirements are not as onerous. This leaves open the question about the circumstances under which scalar models could perform as well as matrix models.

Choice of model structure, environmental variability, data uncertainty and time series length have independent known impacts in populations risk assessments, but their combined impact has not been thoroughly investigated. Dunham et al. [23] compare matrix and scalar models, yet the study constructs a scalar model using error-free output from a matrix model and then compares the scalar model to this underlying model rather than a comparably constructed matrix model. Here, we expand on Dunham et al. [23] using age-structured population models to simulate population trajectories subject to different controlled levels of variability and uncertainty and then evaluate the predictive capabilities of both scalar and matrix models parameterized with these simulated time series. Thus, we examine the effect of parameterization using error-laden data; a scenario that ecologists are typically confronted with. Since the matrix and scalar models are constructed from the same error-prone data, the output from the two types of models can be comparably assessed. Modeled life-histories in our study are based on a formalism in Dunham et al. [23] as well as that of a rare, threatened snail species. These relatively simple life-histories were constructed to mimic short-lived organisms such as invertebrates, birds and small mammals that are commonly included in conservation threat assessments. We compare the performance of scalar and matrix PVAs constructed from the “observed” data using percent decline, a commonly applied metric in decision-making contexts. We found that scalar models tend to be as reliable as matrix models, and sometimes more so, under environmental variability and data uncertainty when projection horizons are short and life histories are relatively simple, which has implications for population projection studies and conservation decision-making.

## Methods

**Fig 1:**
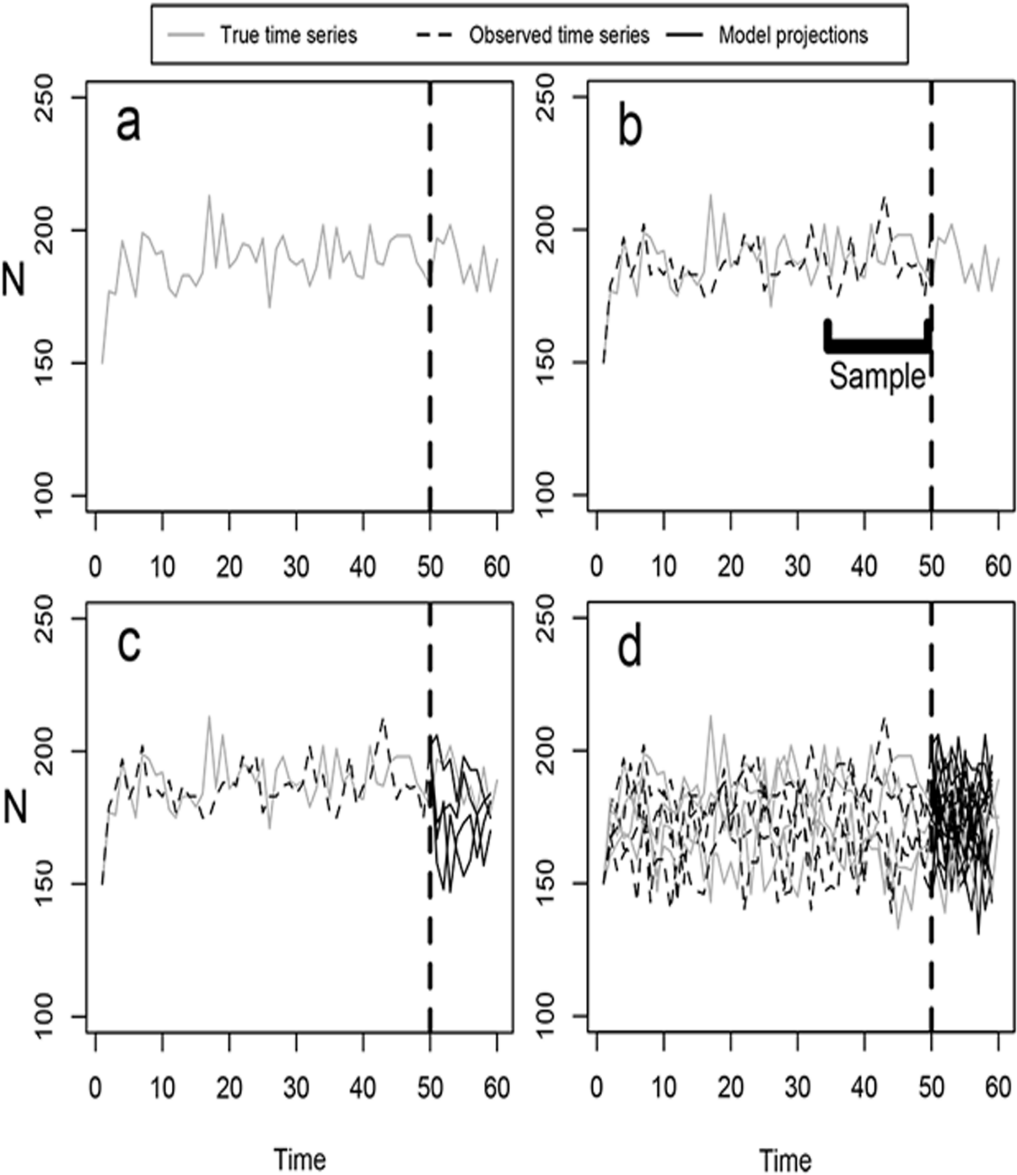
An illustration of the methodology used in this study. a) “True” time series (gray) are generated with underlying process error. b) Measurement error is then added, yielding the “observed” time series (dashed). The “observed” series is then sampled over a specific time period, and these data are used to parameterize matrix and scalar population models, called “estimated” models. c) The “true” series and “estimated” models (black) are projected into the future. The dashed vertical line divides between “past” and projection time frames. d) This process was repeated such that each parameter combination yielded 1000 “true” time series, and each “true” time series led to 1000 replicate projections from both “estimated” matrix and scalar models.

Using age-structured matrix models, we simulated time series population data intended to represent the “true” dynamics of a hypothetical short-lived species and of a rare, threatened snail species, *Tasmaphena lamproides*. The hypothetical life history was a simple one typical of a small mammal or passerine and was constructed to mirror those used in previous modeling efforts [23], while *T*. *lamproides* was included to test whether results hold for an actual species with a different life history from the hypothetical cases. From each of these “true” data sets, we constructed matrix and scalar models and compared population trajectories generated by these to the output of the underlying “true” population given a range of process and measurement error levels, underlying growth rates, and time series lengths (Fig 1). The methods in our study specifically included three steps: 1) simulating sets of “true” and “observed” population time series data for both the hypothetical life history and for *T*. *lamproides* with age-structured matrix models under a range of growth rates, levels of process error and measurement error, 2) constructing scalar and age-structured models from a range of time series lengths in the “observed” data; and 3) comparing projections from the PVA models with population trends in the underlying “true” data over the same time period.

### Simulation of “true” and “observed” time series

We based our methods and hypothetical life history structure on the study presented in Dunham et al. [23], modified and expanded to include a range of growth rates (*λ*), process error, and measurement error. For both the hypothetical life history and *T*. *lamproides* we simulated sets of “true” population time series data using age-structured, density-independent stochastic matrix models, *n*_*t*+1_ = *A*_*t*_ *n*_*t*_, where *n*_*t*_ is the age-specific population vector and *A*_*t*_ is the population projection (or Leslie) matrix. We focused on density-independent models throughout for two important reasons: 1) we wished to investigate the effects of process error, measurement error, and data length on modeling declining populations that are less likely to be driven by density dependence, and 2) we wished to compare and contrast our results with those in key papers which also do not include density dependence [23, 25]. As in Dunham et al. [23], our simulated “true” population for the hypothetical life history had ten age classes, while for *T*. *lamproides* the simulated “true” population had 5 age classes as in Regan et al. [26]. Unlike Dunham et al. [23], who investigated the effect of generation length on quasi-extinction risk estimates, we always set the age of first reproduction at one—only one of the generation length scenarios tested in Dunham et al. [23].

We chose four population growth rates (0.9, 0.95, 1.0, 1.025) and four levels of process error (0, 0.1, 0.3, 0.5; however for *T*. *lamproides* the highest value was 0.45 instead of 0.5, see below) modeled as a co-efficient of variation in the vital rates (Table 1). For both life histories, a population projection matrix was assembled for each combination of *λ* and level of process error (Table 2). To ascertain the effects of process error, measurement error, and data length on populations for which a PVA could be illuminating, we chose growth rates 0.9 ≤ *λ* ≤ 1.025. Because we were interested in investigating the effects of variability and uncertainty on rates of population decline, and not on rates of increase, we narrowed our focus to these growth rates because they could exhibit declines under environmental stochasticity without projecting populations that always declined to zero. Levels of process error were chosen to correspond to ranges of variability documented in natural populations [15, 26].

**Table 1:** Parameter values used in simulations.

Parameter	Value
Growth rate	0.9, 0.95, 1.0, 1.025
Vital rate (CV)	0, 0.1, 0.3, 0.45^a^,0.5
Measurement error (CV)	0, 0.1, 0.3
Data length sampled (*M*)	5, 10, 15, 20, 30, 40, 50

A total of 336 parameter combinations (referred to as scenarios) were considered.

^a^A coefficient of variation of 0.45 instead of 0.5 was used to generate “true” sets of data for *T*. *lamproides*.

**Table 2:** Projection matrix for the hypothetical life history.

Age Class	1	2	3	4	5	6	7	8	9	10+
**1**	*f*	*f*	*f*	*f*	*f*	*f*	*f*	*f*	*f*	*f*
**2**	0.5	0	0	0	0	0	0	0	0	0
**3**	0	0.5	0	0	0	0	0	0	0	0
**4**	0	0	0.5	0	0	0	0	0	0	0
**5**	0	0	0	0.5	0	0	0	0	0	0
**6**	0	0	0	0	0.5	0	0	0	0	0
**7**	0	0	0	0	0	0.5	0	0	0	0
**8**	0	0	0	0	0	0	0.5	0	0	0
**9**	0	0	0	0	0	0	0	0.5	0	0
**10+**	0	0	0	0	0	0	0	0	0.5	0.5

Values in the first row represent fecundities (*f*). Fecundities were back calculated so that the dominant eigenvalue of the resulting matrix was equal to the *a priori* specified growth rate (λ). Therefore, fecundities vary per growth rate (λ). A given fecundity value was assumed to be the same across ages. Fecundity when λ = 0.9 was 0.4, fecundity when λ = 0.95 was 0.45, fecundity when λ = 1.0 was 0.5 and fecundity when, λ = 1.0250 was 0.5250. The off-diagonal values represent the survival rates.

In the case of the hypothetical life history, mean survival rates were held constant at 0.5 across all age classes and for all combinations of growth rate and process error, as in Dunham et al. [23]; this was considered realistic because an average survival rate of around 0.5 is commonly observed in passerines [27] and small mammals [28], organisms that also exhibit relatively simple life histories. The expected probability of an individual surviving to age 10 in this model is approximately 0.001, however the range of process error implemented across the scenarios in Table 1 can give much higher actual probabilities. In the case of *T*. *lamproides*, survival rates followed Regan et al. [26] and were as follows: 0.4 for age class 1, 0.5 for age class 2, 0.8 for age class 3, 0.75 for age class 4, 0.7 for age class 5 (Table 3). Here, survival rates were different across age classes, with some of them being higher than the value used in the simulation of the hypothetical organism (0.5). Since these average survival rates were generally higher than those implemented in the hypothetical model described above, the coefficients of variation for process error in vital rates in the *T*. *lamproides* model were restricted in order to ensure stochastic survival rates generally fell between 0 and 1.0 (Table 1).

**Table 3:** Projection matrix for *T. lamproides*.

Age Class	1	2	3	4	5+
**1**	*f*	*f*	*f*	*f*	*f*
**2**	0.4	0	0	0	0
**3**	0	0.50	0	0	0
**4**	0	0	0.8	0	0
**5+**	0	0	0	0.75	0.7

Values in the first row represent fecundities (*f*). Fecundities were back calculated so that the dominant eigenvalue of the resulting matrix was equal to the *a priori* specified growth rate (λ). Therefore, fecundities vary per growth rate (λ). A given fecundity value was assumed to be the same across ages. Fecundity when λ = 0.9 was 0.3292, fecundity when λ = 0.95 was 0.3976, fecundity when λ = 1.0 was 0.4630 and fecundity when, λ = 1.0250 was 0.4947. The off-diagonal values represent the survival rates.

For both life histories simulated, and as in Dunham et al. [23], average fecundities were assumed to be the same across breeding age classes, and were inversely calculated so that the dominant eigenvalue of the resulting matrix was equal to the *a priori* specified growth rate in each scenario (Tables 2 and 3). Average vital rates, together with specified levels of process error, were used to parameterize distributions from which realized year-to-year vital rates were drawn. Stretched beta distributions, bounded at 0 and 1000, represented process error in fecundities, while beta distributions on the interval [0,1] represented process error in survival rates [10]. Vital rates were sampled randomly and independently from these distributions to provide a population projection matrix of uncorrelated vital rates in each time step. While the use of correlated vital rates is advised to provide a conservative estimate of risk [29], we opted to keep vital rates uncorrelated for methodological simplicity and interpretability of results.

The initial population size was set to 1 × 10^9^ to avoid excessive extinctions, and assumed to be at a stable age distribution. The assumption of an initially stable age distribution was adopted to avoid falsely attributing population fluctuations resulting from age transitions to process error, as our aim is to determine the effect of process and measurement error on modeled population dynamics and not the effect of deterministic fluctuations due to population age structure. A stable age distribution is also a common assumption in PVA applications where initial abundance is uncertain or unknown [29]. In line with Dunham et al. [23] and Wilson et al. [25] we did not include demographic stochasticity, which is negligible in large populations.

Code was constructed in Matlab 7.5.0 [30]. We ran each simulation for 60 years: the first 50 years provided the “true” time series data from which “observed” time series of different lengths were sampled, whereas the last 10 years served as the projection of the “true” population dynamics for comparison with the models based on “observed” data. To capture the effect of process error, we simulated 1000 “true” population time series for each combination of parameter values in Table 1. Populations never fell below 1000 individuals in the first 50 years of a simulation, regardless of the life history, vital rate C.V., or growth rate considered, meaning that our omission of demographic stochasticity did not introduce any bias into our results.

For each of the 1000 trajectories generated from each combination of growth rate and process error, we created a related “observed” time series by adding measurement error to each age-specific abundance in the “true” time series. Measurement error was assigned independently by sampling from a Normal distribution with the “true” abundance as the mean and the respective coefficient of variation for measurement error providing the standard deviation (Table 1), bounded below by zero. We assigned three different levels of measurement error using coefficients of variation 0.0, 0.1 and 0.3 (Table 1). This range of measurement error is somewhat smaller than in other studies [25,31], yet likely reflects the upper range in real population census data [13]. Seven different lengths of annual “observed” time series data were then taken from the last *M* years of the 50 year simulated population sizes (either age-based or aggregate), where *M* is a time series length in Table 1. This resulted in 336 unique combinations of growth rate, process error, measurement error, and time series length, henceforth referred to as scenarios. Each scenario was replicated 1000 times.

### Construction and projection of estimated PVA models

We parameterized stochastic scalar and matrix models from the “observed” time series data from each scenario, henceforth called “estimated” models. Scalar and matrix PVAs were constructed from the 336 time series scenarios. For the scalar models, *M*– 1 stochastic growth rates were calculated as $\frac{n_{t+1}}{n_{t}}=\lambda_{t}$, where *n*_*t*_ is the total observed population size at time *t*. The matrix models were constructed with nine age classes for the hypothetical life history and with four age classes for *T*. *lamproides*; the final stage in each life history was a composite class that included all individuals 9 or 4 years (respectively) and older [32]. For each age class except the composite class, survival rates were calculated as $\frac{n_{i+1,t+1}}{n_{i,t}}$, where *n*_*i*,*t*_ is the observed population size in age class *i* = 1…8 and time *t*. The survival rate for the composite age class was calculated as $\frac{n_{10,t+1}}{n_{9,t}+n_{10,t}}$. Fecundities were assumed to be equal across the age classes and calculated as $\frac{n_{1,t+1}}{\sum_{i=1}^{9}n_{i,t}}$.

To avoid introducing a potential bias associated with specifying a particular distribution, process error in vital rates (growth rates in the scalar model and fecundities and survivorships in the matrix model) was introduced by sampling vital rate values calculated from the “observed” series directly, with replacement, for each time step in the projection of the “estimated” models. Thus, there were *M*– 1 vital rates that could be sampled from for a given “observed” series, leading to a distribution of actualized rates in the “estimated” projection. In some cases, large measurement error caused “observed” populations in an age class to be higher than that of the prior age class in the prior year, making the calculated survival rate larger than one; these estimated survival rates were not included in the projected sampling.

We next projected 1000 population trajectories for ten years for each of the 1000 “estimated” scalar and matrix models per scenario. The projection length was selected to coincide with the IUCN Red List criterion A.3 for declining populations, which evaluates population declines “projected to be met in the future over a time frame of either 10 years or 3 generations, whichever is longer (up to a maximum of 100 years)” [4]. Three generations in our models was always less than ten years. In each future projection, the initial population size was set as the final age-based or total population size in the “observed” time series on which the model was based. While this time horizon is relatively short, it represents the appropriate scale for projections of short-lived species who are often the most appropriate candidates for PVAs.

### Comparison of “true” and “estimated” projections

The median percent population decline was calculated for each scenario and compared to the “true” population’s decline projected over the same period. The median percent decline was chosen to represent a moderate (precautionary but realistic) attitude to variability and uncertainty in calculating declines from stochastic population models, as recommended in the guidelines for using the IUCN Red List criteria [4]. This resulted in 1000 comparisons of medians from “estimated” model output with a corresponding “true” decline for each parameter combination scenario, depicted as the difference between the “true” and “estimated” decline. When comparing “true” and “estimated” model output, we focused on both *precision* and *bias*. Precision was measured by the inverse of the width of the interquartile ranges of percent decline differences, with small ranges indicating a high degree of reproducibility—and therefore precision—in the “estimated” models. Bias was examined as the tendency of differences in “true” and “estimated” percent declines to be centered at zero, with differences closer to zero providing more unbiased predictions.

## Results

**Fig 2:**
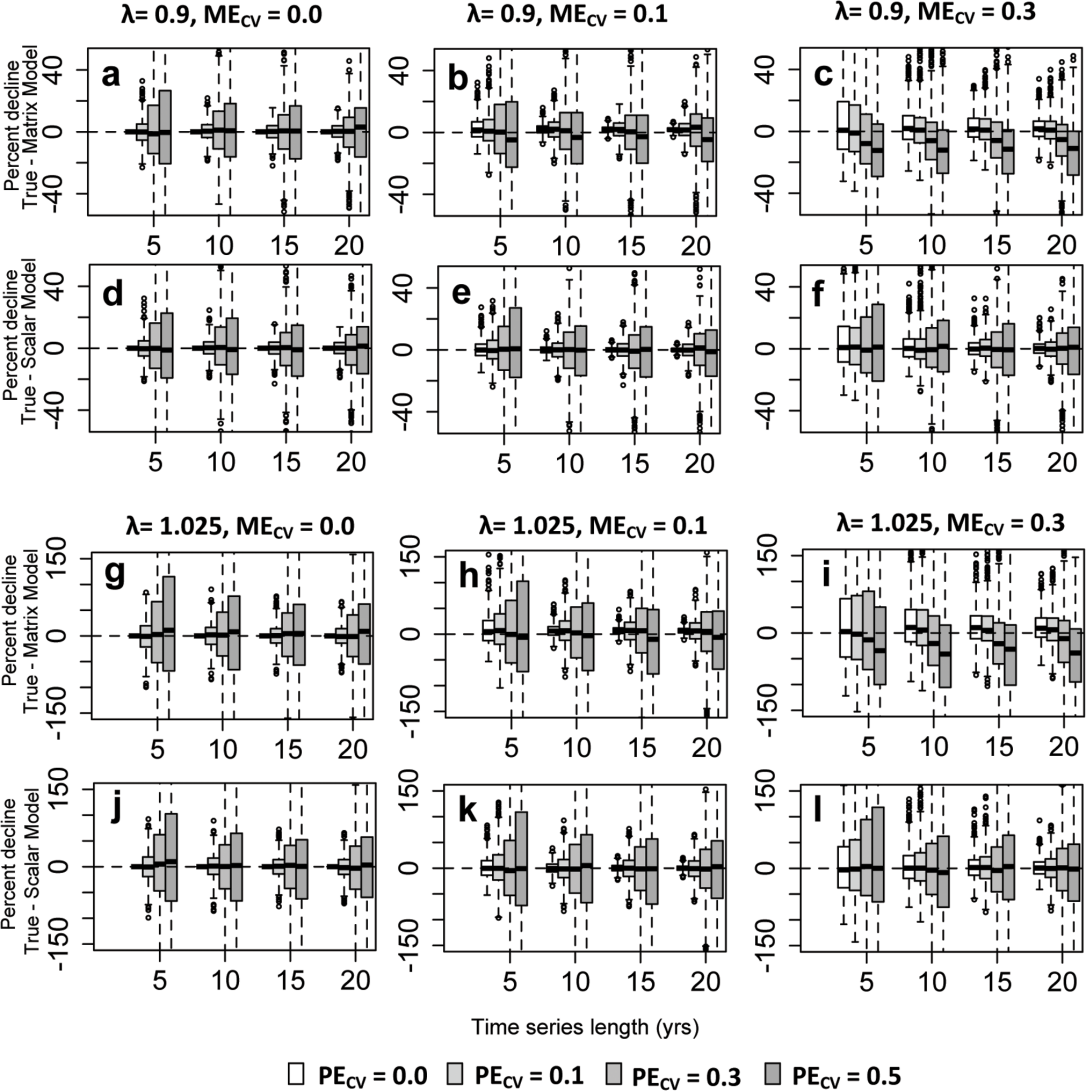
Box and whisker plots showing the difference in percent decline between the “true” and median “estimated” model projections for the hypothetical life history. Boxes are centered about the median difference while the box extension covers the interquartile range. a-c) Matrix model, *λ* = 0.9. d-f) Scalar model, *λ* = 0.9. g-i) Matrix model, *λ* = 1.025. j-l) Scalar model, *λ* = 1.025.

**Fig 3:**
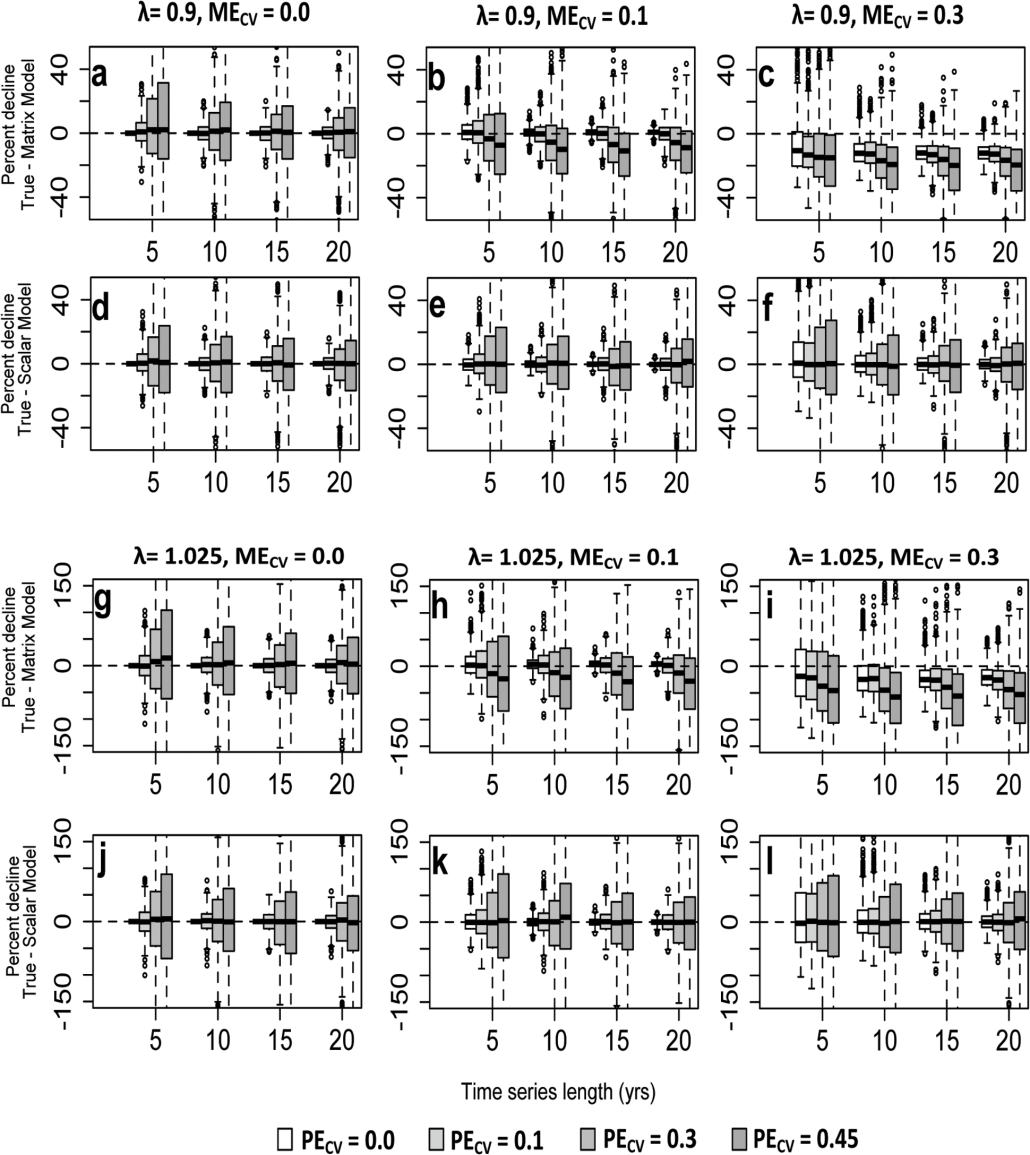
Box and whisker plots showing the difference in percent decline between the “true” and median “estimated” model projections for *T*. *lamproides*. Boxes are centered about the median difference while the box extension covers the interquartile range. a-c) Matrix model, *λ* = 0.9. d-f) Scalar model, *λ* = 0.9. g-i) Matrix model, *λ* = 1.025. j-l) Scalar model, *λ* = 1.025.

Realized stochastic growth rates for the true models behaved as expected over the ten year projection period, with the stochastic growth rate decreasing and the standard deviation increasing as process error increased (S1 Table). There were no observable differences in stochastic growth rates between the hypothetical and snail life histories. When the true series were compared to simulated time series via percent declines, more complex patterns emerged. Independently, increasing measurement and process error and decreasing the length of time series used to parameterize “simulated” models generally reduced the precision and increased the bias of predicted percent declines. These effects were apparent across hypothetical and snail life histories, models, and scenarios considered. Across scenarios, process error tended to increase the spread of predictions more than measurement error (Figs 2 and 3; S1 and S2 Figs), while both contributed strongly to bias. However, the magnitude of effects of measurement error, process error, and time series length, and interactions between these, depended on context. We highlight the most notable of these results below.

### Precision of projections of decline for the hypothetical life history

The effect of time series length on the precision of percent decline estimates for the hypothetical life history was similar across scalar and matrix models but differed across growth rates, with time series length having a greater impact on precision when growth rates were high (i.e., 1.025 and 1.0; Fig 2G–2L, S1G–S2L Fig). Increasing time series length discernibly decreased the width of the interquartile ranges of percent decline differences under the full suite of process error C.V.s. However, marked increases in precision only occurred up to a time series length of around 10 to 15 years, beyond which the precision of simulated percent declines did not notably improve in the short projection span we considered (Fig 2, S1 Fig). The greatest improvement in precision due to time series length consistently occurred in the increase from 5 to 10 years. For example, when *λ* = 1.025, ME_CV_ = 0.3 and PE_CV_ = 0.3, the interquartile range of the difference in percent decline for matrix and scalar models decreased by ~36% and ~33%, respectively, as data length increased from 5 to 10 years. In contrast, the relative reduction in the interquartile range for matrix and scalar models decreased only by ~16% and ~12% respectively when data length went from 10 to 15 years for the same parameter combination. Although present, further changes in data length after 15 years were even much smaller in magnitude (Fig 2 *I*).

While measurement error generally reduced the precision of percent declines across scenarios considered, having longer time series consistently improved the precision of predictions when measurement error was present. The strongest beneficial effects of increasing time series length in the face of measurement error appeared with higher levels of process error and again with an increase from 5 to 10 years of data (Fig 2C, 2F, 2I and 2L, S1C, S1F, S1I and S1L Fig). For instance, when the growth rate was *λ* = 0.9, the interquartile range of decline differences for *M* = 10 and ME_CV_ = 0.1 was narrower than that for *M* = 5 and a ME_CV_ = 0 when PE_CV_ > 0; this was true for both scalar and matrix models (Fig 2A, 2B, 2D and 2E). Likewise, when the growth rate was 1.025, the interquartile range for *M* = 15 and ME_CV_ = 0.3 was narrower than that for *M* = 10 and ME_CV_ = 0.1 for large PE_CV_ (Fig 2H, 2I, 2K and 2L). This effect was similar regardless of the model used, but had a greater magnitude for larger growth rates 1.0 and 1.025.

### Biases in projections of decline for the hypothetical life history

The bias in estimated percent declines depended on the model chosen, the growth rate, and the levels of process and measurement error in the data. Bias in estimated declines did not appear to depend appreciably on time series length (Fig 2, S1 Fig). Declines estimated with scalar models did not exhibit noticeable bias, irrespective of the growth rate, PE_CV_ or ME_CV_ (Fig 2D–2F and 2J–2L, S1D–S1F and S1J–S1L Fig). However, percent decline differences estimated with matrix models exhibited a bias, the direction and extent of which was dependent on the combination of growth rate, PE_CV_ and ME_CV_ (Fig 2A–2C and 2G–2I, S1A–S1C and S1G–S1I Fig). In general, as the overall variability in vital rate parameter estimates increased due to a confluence of PE_CV_ and ME_CV_, predictions progressed from reasonably unbiased, to under-estimation, and then to over-estimation. This pattern became more pronounced as growth rates increased from 0.90 to 1.025. Overestimation was generally observed in scenarios with both high process and high measurement error (Fig 2C and 2I). Underestimation tended to occur at lower measurement error and low to moderate process error (Fig 2A, 2B, 2G and 2H). In general, overestimation biases were greater in magnitude than underestimation biases.

### Precision and bias of projected declines for *T*. *lamproides*

Generally, the overall performance of the two models across the different scenarios evaluated exhibited the same pattern for both of the life histories studied. However, results for *T*. *lamproides* departed from the hypothetical life history in a few key ways. We focus below on highlighting these important departures from the overall patterns seen in the hypothetical life history below.

Precision was slightly higher in *T*. *lamproides* than in the hypothetical life history across scenarios with high levels of measurement error for both matrix and scalar models. However, precision in scalar models exhibited smaller differences between the life histories relative to matrix models. For example, the interquartile range of decline difference for *T*. *lamproides* was ~40% smaller than that of the hypothetical life history for matrix models, yet only 20% smaller for scalar models, when the growth rate was *λ* = 1.025, M = 5, ME_CV_ = 0.3 and PE_CV_ = 0.3 (Figs 2I and 3I).

The bias observed in projected percent decline in the matrix model for *T*. *lamproides* was much more unidirectional towards overestimation. The magnitude of this bias intensified when measurement error increased, and was observable even in scenarios not exhibiting bias under the hypothetical life history (compare, e.g., Figs 2 and 3. c, i, S1 and S2 Figs c,i). However, as was the case with the hypothetical life history, higher growth rates intensified the overestimation bias.

## Discussion

Our results suggest that the least biased estimates of projected declines are obtained when measurement error is minimized irrespective of time series length. In contrast, longer time series did improve the precision of projections, although it had diminishing benefits beyond 10–15 years. We also found that scalar models can project percent declines quite well relative to matrix models for short-lived organisms with simple life histories, like the ones studied here, given their comparable precision and less biased predicted declines. This is an encouraging result given the ease with which scalar models can be parameterized and implemented.

Measurement error, process error, time series length, and model choice all affected the ability of PVAs to reliably project viability, although at times in unanticipated ways. As expected, high levels of process and measurement error reduced the reliability of the two models’ projections, with process error tending to contribute to an increase in the spread of predictions more than measurement error in percent decline projections (see also [13,19]). Bias tended to be low for both models except in some cases when measurement and process error became large, where matrix models tended to over-estimate percent declines. This over-estimation was amplified as growth rate increased. For these instances, high bias was not ameliorated by longer time series, as it was largely a consequence of the parameterization scheme. In contrast, both lower measurement error and longer time series tended to generate more precise projections for both models, but lowering measurement error had more consistently observable effects across all scenarios.

The percent population decline calculated here uses a central tendency measure (the median of population trajectories, as recommended in [4]) to characterize “estimated” trajectories. This metric is a valuable indicator of population trajectories under risk and uncertainty; for instance, it can be interpreted as a “best estimate” in the IUCN Red List criteria for classifying extinction risk status of taxa. Given the disparity in model performance assessed by percent decline, we recommend looking at the effects of model type, process error, and measurement error on extinction risk classification using a decision rule framework. Matrix models may have additional benefits in such a framework because they allow the sensitivity of projections and subsequent classification to changes in age/stage-specific vital rates to be examined. Still, a key unresolved question from our and other studies is to what extent percent decline differences translate into over-protection or under-protection errors, and whether these tend to be substantial for particular combinations of measurement error, process error, or model type. Reducing errors in prioritization and decision making is paramount in conservation biology where resources are extremely limited: over-protection errors can lead to placing high conservation priority on a taxon when it is not warranted whereas under-protection errors can lead to taxa not receiving the conservation intervention they require for persistence. Studies have evaluated the effects of variability and uncertainty on classification accuracy using different decision rule systems [25, 33], although none to our knowledge have compared scalar and matrix model types in such an analysis.

Surprisingly, time series length did not appear to be particularly influential on model performance when process error and measurement error were present after 10–15 years of data were included. As observed elsewhere, we found that the precision of the projections increased as data length increased [12,19]. However, biases remained as time series length increased, and the benefits of increasing data length disappeared after 20 years of data. This falls within the range of data used by Meir & Fagan [13] and are consistent with McCarthy et al. [34] and Humbert et al. [20] who suggest that a 10-year time series is sufficient to inform management decisions (but see [19]). However, our projection period was short, reflecting the rapid generation times of our modeled life-histories; declines projected over longer periods may require longer time series to exhibit similarly high level of precision and low bias we observed here ([35]). In particular, it is likely that the benefits of longer time series may not diminish over similar time scales, and may not decline at all over realistic lengths for very long-lived species requiring multi-decadal projection lengths.

Regardless, the reduced benefits we observed with longer time series indicates the effects of measurement and process error cannot be eliminated with longer time series length, especially with regards to model bias. Rather, our results suggest that it is better to invest in more sophisticated sampling techniques (e.g. [36]) or parameterization methods (e.g. state-space models; see below) that can reduce or quantify measurement error than to rely on longer census periods. Additionally, analyses of marked individuals can provide more robust estimates of survival [36,37] that can be used in lieu of, or in addition to, estimates derived from time series. This is an added benefit of the fact that marking individuals is likely necessary in most systems to obtain ages of individuals with a reasonable level of certainty.

Matrix models mostly exhibited a precautionary tendency (i.e. over-estimation) when estimating percent decline. While our observed over-estimation tendency is similar to that presented for scalar models in Dunham et al. [23], we also observed the same patterns in under- and over-estimation for “estimated” matrix models. A similar construction of matrix models from “observed” data was absent from Dunham et al. [23] and hence they concluded matrix models are less conservative than scalar models. Dunham et al. [23] focused on quasi-extinction risk rather than percent decline; these two metrics may not exhibit exact similarities in their sensitivities to measurement and process error. However, our results do concur with others that have found an over-estimation tendency of extreme decline among PVA methods [25] as well as those that suggest that simple models can perform very well relative to matrix models for population projections, especially when uncertainty is high [38].

The strong over-estimation bias observed in percent declines generated by matrix models is due to the way in which the models were parameterized. With elevated levels of process and measurement error, there were instances in which the measurement error increased the “observed” abundance of a cohort from one year to the next, generating age-based survival rates greater than one. Since survival rates greater than unity are not biologically possible, any calculated as such were discarded and not used in the “estimated” projections. Although this constrained the age-based survival rates to realistic values, it also biased the predictions by increasing the sampling of lower survival rates. The overrepresentation of lower survival rates in turn resulted in model projections with lower realized population growth rates. This bias became more pronounced as the input growth rate of the “true” population increased. As a result, populations in decline, such as threatened populations, were projected with greater precision and less bias using matrix models than growing or stable populations, (see also [25]). Not censoring unrealistic survival rates in the simulated matrix models would have potentially generated an underestimation bias (this result was confirmed in preliminary simulations; results not shown). This effect is similar to that seen for matrix models under lower levels of process error; here, survival rates inflated by measurement errors lead to realized growth rates in “estimated” projections that exceed those for the true population, especially when the “true” growth rate was already high. For scalar models, our parameterization technique appears more robust. The only vital rate estimated in scalar models is the geometric growth rate, which was not truncated and—unlike survival rates—could increase without constraint. Thus, the fact that these biases weren’t observed in scalar model projections is attributable to the simplicity of the model structure.

The role of measurement error in biasing matrix model predictions—an effect that remained when long time series were used—highlights the importance of dealing with this source of uncertainty when parameterizing PVAs. Our parameterization method was inspired by examples given in widely used conservation biology textbooks (although without using their precise formulations), combining calculations for survival and fecundity from Akçakaya et al. [32] and sampling from these observed rates to parameterize projections as implemented in Morris and Doak [10], although the latter does not address parameterization from time-series nor do either source address the conundrum of unrealistic survival estimates. Although ad-hoc, our choices were partially based on a desire to avoid biases introduced by specifying particular distributions—opting again for simplicity and transparency—as well as mimic what a “first pass” parameterization effort might look like in a basic PVA implementation. Neither these simple methods nor related techniques account for measurement error and, in our case, still introduced substantial biases at high levels of measurement and process error. There are, of course, other methods available for parameterizing PVAs. State space models in particular are an effective way to take into account measurement error when assessing population viability [18,25]. While state space methods are technically challenging, data hungry (i.e. require long time-series), and difficult to apply to non-linear problems, they do appear desirable to avoid biases when both variability and uncertainty are high. When not employing methods that explicitly account for measurement error, using simpler scalar models, or at least comparing outcomes generated by alternative parameterizations of matrix and scalar models, may be a wise alternative because of lower data requirements.

We assumed simple life histories possessing short generation times with additional simplifications that vital rates were not correlated, were independently sampled from identical distributions, and that population dynamics lacked density dependence. Relaxation of these simplifications could alter the way in which variability and uncertainty interact. For instance, density dependent models such as the Ricker model are highly susceptible to the negative effects of measurement error [13,19]. Other types of variability such as demographic stochasticity and heterogeneity may differentially affect certain life stages and interact with density-dependence (e.g. [39]). Correlations in vital rates among life stages are likely in species with relatively simple life histories; including these could also alter our results, as correlations can increase the variability, and thus extinction risk, of a population [10,29]. Not including correlations in the “estimated” matrix models if they were included in the “true” model would therefore probably exacerbate the bias and reduced precision we observed. Scalar models, in contrast, may be relatively immune to this effect since they are parameterized using observed variability across the entire population. While the effect of environmental correlation of vital rates has been shown to be influential on model results in some studies (e.g. [40]), in others it has shown to have negligible effects (e.g. [41]), and requires additional data and choices in model construction to implement. Comparing how different forms and strengths of correlated variability interact with the factors we examine in the present study holds exciting potential for future research.

In addition, different life history traits could have dramatically different impacts on the reliability of the models evaluated here. We observed such differences in our study between the hypothetical and *T*. *lamproides* life histories. For *T*. *lamproides*, precision of matrix models increased but bias also increased relative to the hypothetical life history, whereas the precision and bias for scalar models between these remained roughly the same. Because *T*. *lamproides* possessed fewer life stages than the hypothetical life history, uncertainty was compounded to a lesser extent and precision increased. In contrast, higher survival rates in most *T*. *lamproides* life stages led to increased bias, as the biasing effects generated by the matrix model parameterization described earlier in the discussion was intensified.

Despite their differences, both life histories we examined had short generation times (<10 years across all scenarios) with breeding possible in the first life stage. The assumptions we have made are reasonable for many organisms including longer-lived invertebrates, birds, and small mammals. However, Dunham et al. [23] observed that over-estimation bias in extinction risks estimated with scalar models increased as generation time increased due to misattribution of deterministic fluctuations to stochastic variability, highlighting the sensitivity of modeling methods to life history features. While some simple statistical models of extinction risk may be robust to commonly included model complexities such as density dependence [38], a more extensive evaluation of a broad range of life history traits is needed in order to determine when scalar or matrix models will perform better for a particular life history under process and measurement error. Our results do suggest however that in those cases where matrix models may be clearly desirable, such as when organisms have very complex life histories, care still needs to be used in their application for projecting population abundances when variability and uncertainty are high.

## Supporting Information

S1 Code Model code used to generate simulation results. Model files are *.m files that require Matlab to run. The “main” file is runMe.m; all other files are called by this main file.
(ZIP)

S1 Dataset Model simulation output. Files are organized by model type and by the input lambda value.
(ZIP)

S1 Fig Box and whisker plots showing the difference in percent decline between the “true” and median “estimated” model projections for the hypothetical life history. Boxes are centered about the median difference while the box extension covers the interquartile range. a-c) Matrix model, *λ* = 0.95. d-f) Scalar model, *λ* = 0.95. g-i) Matrix model, *λ* = 1.0. j-l) Scalar model, *λ* = 1.0.
(TIF)

S2 Fig Box and whisker plots showing the difference in percent decline between the “true” and median “estimated” model projections for *T*. *lamproides*. Boxes are centered about the median difference while the box extension covers the interquartile range. a-c) Matrix model, *λ* = 0.95. d-f) Scalar model, *λ* = 0.95. g-i) Matrix model, *λ* = 1.0. j-l) Scalar model, *λ* = 1.0.
(TIF)

S1 Table Average stochastic growth rates for the “true” models across scenarios. Stochastic growth rates are calculated as geometric means from each time series over the ten year projection period. Averages of the stochastic growth rates reflect arithmetic means.
(DOCX)
