# Supplementary figures and images for: Combined Influences of Model Choice, Data Quality, and Data Quantity When Estimating Population Trends
Source: PLoS One. 2015 Jul 15;10(7):e0132255. doi: 10.1371/journal.pone.0132255 (PMC4503393; doi:10.1371/journal.pone.0132255)

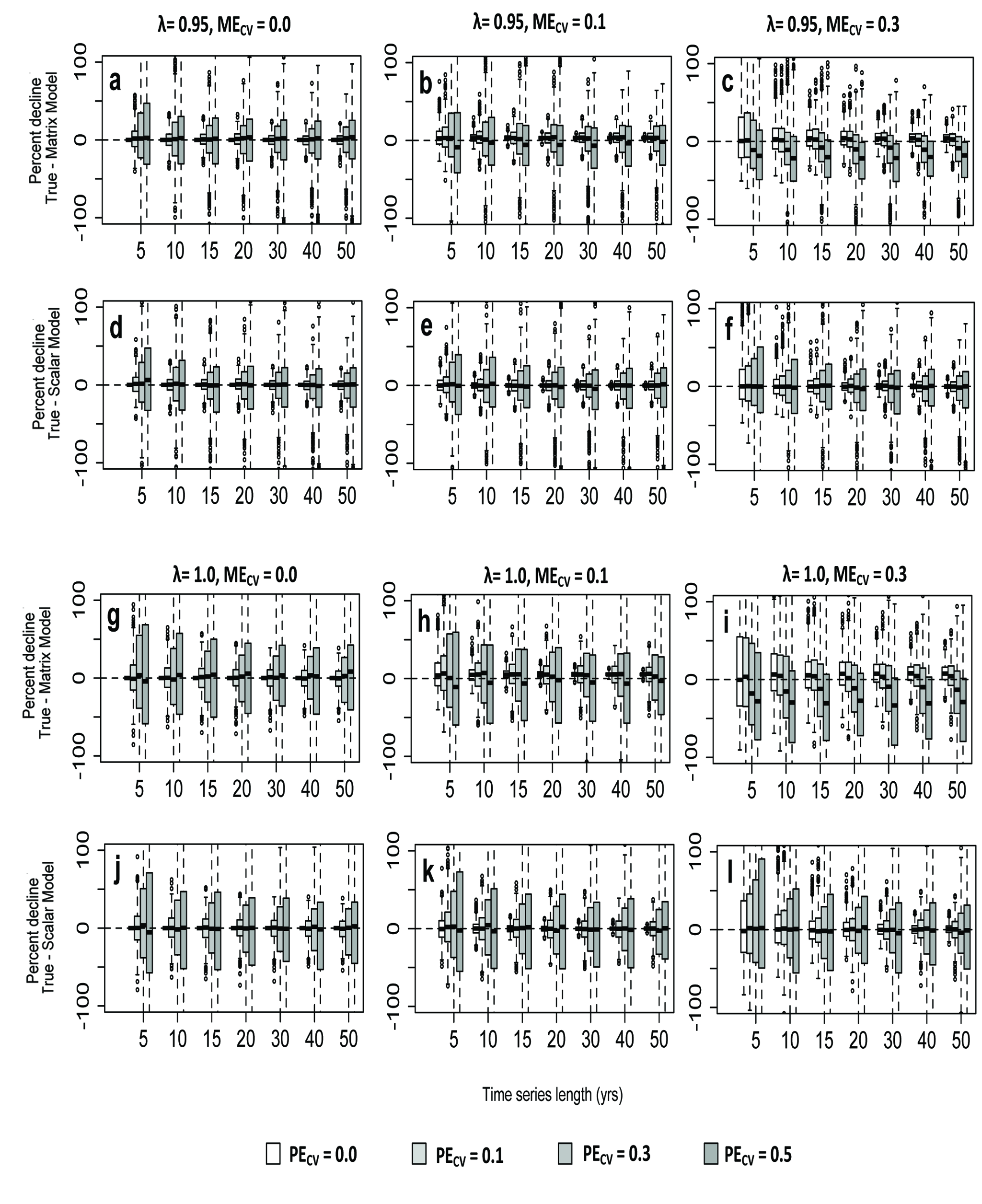

Supplement: S1 Fig — Boxes are centered about the median difference while the box extension covers the interquartile range. a-c) Matrix model, λ = 0.95. d-f) Scalar model, λ = 0.95. g-i) Matrix model, λ = 1.0. j-l) Scalar model, λ = 1.0. (TIF) [file pone.0132255.s003.tif]

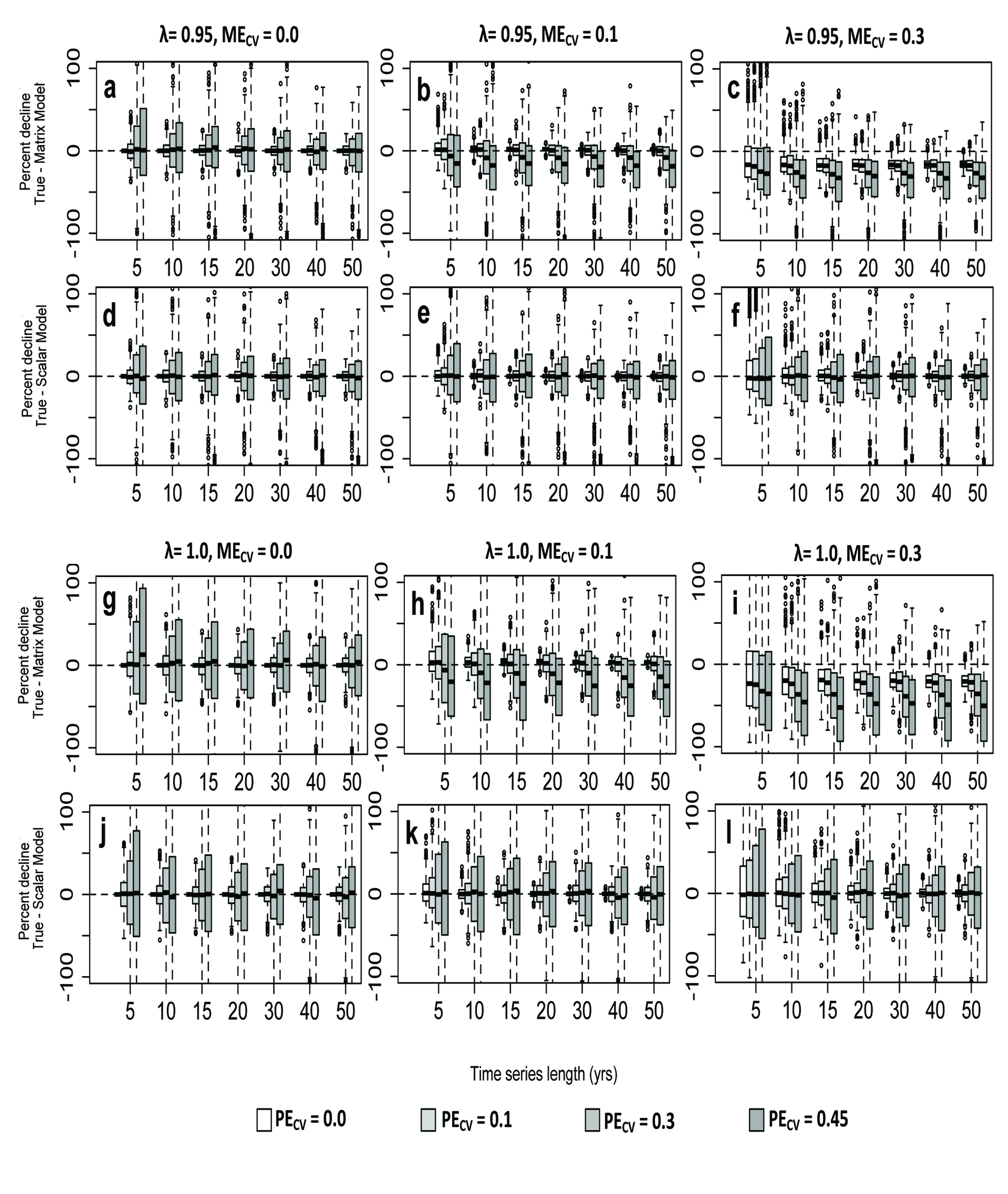

Supplement: S2 Fig — Boxes are centered about the median difference while the box extension covers the interquartile range. a-c) Matrix model, λ = 0.95. d-f) Scalar model, λ = 0.95. g-i) Matrix model, λ = 1.0. j-l) Scalar model, λ = 1.0. (TIF) [file pone.0132255.s004.tif]
