# Supplementary material for: Combined Influences of Model Choice, Data Quality, and Data Quantity When Estimating Population Trends
Source: PLoS One. 2015 Jul 15;10(7):e0132255. doi: 10.1371/journal.pone.0132255 (PMC4503393; doi:10.1371/journal.pone.0132255)
Supplement: S1 Table — Stochastic growth rates are calculated as geometric means from each time series over the ten year projection period. Averages of the stochastic growth rates reflect arithmetic means. (DOCX) [file pone.0132255.s005.docx]

| **Life history** | **Underlying growth rate** | **Process**  **error** | **Average stochastic growth rates** | **Standard deviation** |
| --- | --- | --- | --- | --- |
| **Hypothetical** | 1.025 | 0.5 | 1.006703 | 0.0521313 |
| **Hypothetical** | 1.025 | 0.3 | 1.017024 | 0.0298919 |
| **Hypothetical** | 1.025 | 0.1 | 1.023555 | 0.0096558 |
| **Hypothetical** | 1.025 | 0.0 | 1.025000 | 0.0000000 |
| **Hypothetical** | 1.000 | 0.5 | 0.978947 | 0.0505790 |
| **Hypothetical** | 1.000 | 0.3 | 0.992457 | 0.0286325 |
| **Hypothetical** | 1.000 | 0.1 | 0.998548 | 0.0090903 |
| **Hypothetical** | 1.000 | 0.0 | 1.000000 | 0.0000000 |
| **Hypothetical** | 0.950 | 0.5 | 0.927901 | 0.0463461 |
| **Hypothetical** | 0.950 | 0.3 | 0.944103 | 0.0261314 |
| **Hypothetical** | 0.950 | 0.1 | 0.949400 | 0.0083954 |
| **Hypothetical** | 0.950 | 0.0 | 0.950000 | 0.0000000 |
| **Hypothetical** | 0.900 | 0.5 | 0.882491 | 0.0401884 |
| **Hypothetical** | 0.900 | 0.3 | 0.893082 | 0.0231476 |
| **Hypothetical** | 0.900 | 0.1 | 0.899111 | 0.0077950 |
| **Hypothetical** | 0.900 | 0.0 | 0.900000 | 0.0000000 |
| ***T. lamproides*** | 1.025 | 0.45 | 1.008252 | 0.0400500 |
| ***T. lamproides*** | 1.025 | 0.30 | 1.017645 | 0.0265630 |
| ***T. lamproides*** | 1.025 | 0.10 | 1.023881 | 0.0087976 |
| ***T. lamproides*** | 1.025 | 0.00 | 1.025096 | 0.0000000 |
| ***T. lamproides*** | 1.000 | 0.45 | 0.985206 | 0.0399041 |
| ***T. lamproides*** | 1.000 | 0.30 | 0.995226 | 0.0257786 |
| ***T. lamproides*** | 1.000 | 0.10 | 0.999580 | 0.0079424 |
| ***T. lamproides*** | 1.000 | 0.00 | 1.000000 | 0.0000000 |
| ***T. lamproides*** | 0.950 | 0.45 | 0.938871 | 0.0365754 |
| ***T. lamproides*** | 0.950 | 0.30 | 0.946775 | 0.0237336 |
| ***T. lamproides*** | 0.950 | 0.10 | 0.948748 | 0.0074522 |
| ***T. lamproides*** | 0.950 | 0.00 | 0.950051 | 0.0000000 |
| ***T. lamproides*** | 0.900 | 0.45 | 0.884987 | 0.0348118 |
| ***T. lamproides*** | 0.900 | 0.30 | 0.894259 | 0.0227622 |
| ***T. lamproides*** | 0.900 | 0.10 | 0.899741 | 0.0073687 |
| ***T. lamproides*** | 0.900 | 0.00 | 0.900085 | 0.0000000 |
